# The Influence of Pre-Operative Hormonal Stimulation on Hypospadias Repair

**DOI:** 10.3389/fped.2015.00031

**Published:** 2015-04-22

**Authors:** Nathan C. Wong, Luis H. Braga

**Affiliations:** ^1^Department of Urology, McMaster University, Hamilton, ON, Canada; ^2^Department of Pediatric Urology, McMaster Children’s Hospital, Hamilton, ON, Canada

**Keywords:** androgens, testosterone, hypospadias, urology, surgery, pediatrics

## Abstract

Androgen stimulation to temporarily promote penile growth has been commonly used to facilitate hypospadias repair. Although some series suggest improvement in both functional and cosmetic outcomes, a recent systematic review and meta-analysis showed a possible relationship between pre-operative hormonal stimulation and higher complications. As a result, indications and treatment regimens remain controversial. Here, we review the available literature and present our clinical practice.

## Background

Hypospadias is the second most common male genital birth defect with an incidence of 1 in every 250 male births. Boys with proximal hypospadias typically have small glans penis, making hypospadias repair a technically demanding operation. Repair requires availability of tissue to reconstruct a neourethra of adequate caliber to allow low pressure voiding while minimizing complications including glans dehiscence, urethrocutaneous fistula, meatal stenosis, and unsatisfactory cosmesis secondary to penile scarring. To improve outcomes, use of pre-operative hormonal stimulation (PHS) prior to surgery has been accepted as a relatively common practice among pediatric urologists and surgeons for decades despite the lack of high quality evidence to support it.

Hypoplasia of the penis is believed to make repair more challenging, leading to poorer functional and cosmetic results. Histological studies show that androgens increase the number and density of blood vessels, supporting a theory of neovascularization (1). Observational studies have shown that testosterone (T), dihydrotestosterone (DHT), and human chorionic gonadotropin (hCG) have been associated with a temporary increase in penile length, glans circumference, and tissue vascularity (2–4). These changes have been hypothesized to improve outcomes of hypospadias repair.

Despite widespread use, PHS has been scarcely reported within the literature and outcomes remain controversial. Basic science studies show that androgens impair wound healing and promote inflammation (5). There is also some evidence of potential adverse effects including pubic hair growth, aggressive behavior, accelerated linear height, and bone age. Due to the paucity of high quality evidence, standardized protocols and guidelines are lacking. Herein, we reviewed the currently available literature regarding PHS and present our clinical practice from a tertiary pediatric hospital.

## Surgical Outcomes

Data regarding PHS are mostly from non-randomized studies with limited sample sizes. Koff and Jayanthi reported that hCG significantly increased penile growth, particularly in the proximal region, and moved the meatus closer toward the tip of the glans. They also showed that hCG thickened the corpus spongiosum and decreased chordee severity (6).

In the only randomized controlled trial of 75 consecutive boys, transdermal DHT was shown to decrease complications and improve cosmetic results, including a reduction of penile curvature (7). However, most boys included in this study had mild hypospadias, with 70% of the meatus located at the corona, 24% at the penile shaft, and only 5% at the penoscrotal junction.

On the other hand, a non-randomized cohort study by Gorduza et al. of 126 boys, of whom 30 received PHS, showed that healing complications were higher in the PHS cohort (30%) compared to the non-PHS cohort (17.7%) (*p* = 0.23) (8). This was a small series to reach statistical significance and there was considerable heterogeneity regarding the patients and treatment regimen. Androgen stimulation was also used in more severe cases of hypospadias (penile length <25 mm, hypoplasia and associated undescended testes), which one would expect higher complications regardless of PHS use.

### Systematic review and meta-analysis

In a recent systematic review and meta-analysis by Wright et al. summarizing the effect of PHS on overall complications, a total of 622 patients were included, of whom 283 (45%) received some form of PHS (9). Of the 11 included studies, most were retrospective observational studies of low or moderate methodological quality. Four studies that addressed post-operative complication rates stratified by PHS were included in a meta-analysis that suggested a possible relationship between PHS and increased complication rates in boys with severe hypospadias defects with a non-significant odds ratio of 1.67 (*p* = 0.07).

This finding, however, may have been a result of selection bias within the incorporated studies. Given the subjectivity involved in choosing patients for PHS, it is possible that those selected had more severe anatomical defects with unfavorable tissue characteristic, and consequently carried a greater complication risk. In addition, there was considerable variation and heterogeneity regarding treatment regimen, confounding the results and making them difficult to apply to clinical practice. Another systematic review of 14 studies was performed, including 3 randomized non-blinded studies and 11 non-randomized, non-placebo controlled studies. Similarly, the authors also concluded that the real benefit of PHS on surgical results has yet to be defined (10).

These systematic reviews highlighted that the published literature in this topic is limited and generally of low quality with lack of standardized reporting of important patient and surgical details. Future studies should not only report treatment regimes and technical details, but should also aim to stratify patients according to hypospadias severity, preferably in an objective fashion.

## Indications

Although use of PHS is relatively common, reported indications within the literature are quite subjective and at times lacking. Androgens are typically given in severe cases of hypospadias with the meatus located proximal to the penile mid shaft or with proximal division of the corpus spongiosum (11). Other common indications in the literature include penile length <25 mm, microphallic penile hypoplasia, associated UDT, and reoperations (9). Boys treated with PHS vary in severity from those with meatal location at the distal penile shaft to the perineum. However, there are no clear guidelines available regarding when to use PHS.

A recent study that characterized the practice patterns among mainly North American pediatric urologists via email-based surveys showed that 78% use PHS (12). Androgens were prescribed primarily in small appearing penis, reduced glans circumference, reduced urethral plate width, or proximal hypospadias. However, treatment regimens and definite indications were highly variable.

## Treatment Regimens

Androgen type, delivery mode, dosage, scheduling, and time from PHS to operation are poorly reported in the literature and there is no consensus regarding the details of PHS with lack of data to support one regimen over another. Table 1 summarizes the relevant literature and various hormone regimens. This paucity limits our ability to clearly evaluate various treatment protocols.

**Table 1:** **Hormone regimen, study demographics, and complication rates with and without pre-operative hormonal stimulation**.

Reference	Study design	PHS type (delivery mode)	Dosage	Schedule	Time from PHS to surgery	Reported criteria for PHS	Mean age at surgery (months)	Mean follow-up (months)	Complications [no. patients/total (%)]	
									With PHS	Without PHS
Castañón et al. (13)	Retrospective	DHT (topical)	3%	Daily for 1 month	1 month	All patients received PHS	33	Not stated	1/6 (17)	Not applicable
Koff and Jayanthi (6)	Retrospective	hCG (IM)	250 IU/dose (pts <1 year)	2 times weekly for 5 weeks	6–8 weeks	All patients received PHS	10	Not stated	1/12 (8)	Not applicable
			500 IU/dose (pts 1–5 years)	
Lam et al. (14)	Retrospective	T (IM)	25 mg/dose	1 dose prior to first stage	Not stated	Not stated	34	152	Not stratified	Not stratified
Braga et al. (15)	Retrospective	T (IM)	2 mg/kg/dose	Not stated	Not stated	Subjective	17	35	Not stratified	Not stratified
Braga et al. (16)	Retrospective	T (IM)	2 mg/kg/dose	3 doses 3 weeks apart	Not stated	Subjective	18	36	16/51 (31)	18/86 (21)
Kaya et al. (7)	Randomized	DHT (topical)	2.5% (0.2–0.3 mg/kg)	Daily for 3 months	Not stated	Randomized	33	12	1/37 (3)	9/38 (24)
Catti et al. (17)	Retrospective	T (IM)	100 mg/m^2^	Not stated	2 months	Subjective	27	30	16/26 (62)	Not stratified
de Mattos e Silva et al. (18)	Retrospective	T (IM)	100 mg/m^2^	Not stated	Not stated	Subjective, redo surgery	36	24	23/36 (64)	7/16 (44)
		DHT (topical)	2.50%	Daily for 2 months	
		hCG (IM)	1500 IU	Every 2 days (6 doses)	
Snodgrass and Bush (19)	Retrospective	T (IM)	2 mg/kg/dose	3 doses every 3–4 weeks	Not stated	Subjective	18	12	1/8 (12.5)	2/16 (12.5)
Gorduza et al. (8)	Retrospective	T (IM) hCG (IM) T + hCG (IM)	100 mg/m^2^ 1500 IU 1500 IU	Monthly for 2–6 months	1–24 months	Penis length <25 mm, hypoplasia, undescended testis	36	41	12/30 (40)	23/96 (24)
				Every 2 days (6 doses)	
				Every 2 days (6 doses)	
Rigamonti and Castagnetti (20)	Retrospective	T (IM)	Not stated	2–3 times/month	Not stated	Not stated	16	7	1/9 (11.1)	2/5 (40)

*Adapted from Wright et al. (9)*.

### Type

Several types of PHS are available, with the majority of studies using T compared to DHT and hCG. In a systematic review, 10 studies used T, 2 DHT, 3 hCG, and 1 LH releasing hormone and hCG (10). Some authors feel that DHT may be more effective as it does not rely on 5-alpha reductase activity for conversion of T to DHT. The use of certain PHS varies further depending on geographical availability. Unfortunately, there are no randomized studies in the literature directly comparing T to DHT or hCG to support the use of agent over another.

### Delivery mode

Androgen stimulation may be administered via various routes including topically and intramuscularly. While T, DHT, and hCG are commonly administered by the intramuscular route, DHT and T may be given topically and are found to provide high concentrations. In contrast to the topical route, which is associated with variations in serum T levels, the systemic routes allows for more reliable serum levels (21–23). Topical use also demands parental compliance for the child to receive appropriate dosages (22). Regardless, randomized studies directly comparing topical and intramuscular androgen administration do not show any differences in penile growth between the routes (4, 21). In the systematic review, intramuscular administration seemed to have fewer adverse events than topical (10). In particular, it was noted that topical administration was associated with more skin irritation and genital pigmentation.

### Dosage and schedule

Typical doses and scheduling of androgens vary within the literature. Some authors use intramuscular T doses adapted to patient’s size with substantial effects on penile growth by using doses of 2 mg/kg/dose for two to three doses (2, 15, 19, 24) or 100 mg/m^2^ (8, 18). Alternatively, fixed doses of T at 25 mg/dose IM once monthly for 3 months have also been used with no significant adverse effects such as delay in bone age (11, 14). Similar effects can be achieved with topical 10% T propionate cream applied twice daily (23). If topical DHT is used, its concentration typically ranges from 2.5 to 3% applied daily for up to 3 months (7, 18). While hCG may be administered at 250 IU/dose in patients younger than 1-year-old or 500 IU/dose in patients 1–5 years old, it is typically given as 1500 IU every 2 days for a total of six doses (6, 8, 18).

### Time from PHS to operation

When reported, the time from PHS to operation ranged from 2 weeks to 24 months (2, 8, 24). Some studies skipped doses if certain measures were achieved such as notable increases in penile length or glans circumference (11). It has been reported that serum T levels return to normal range a week after being stopped and penile growth disappears during the first year following treatment (22, 24). Gorduza et al. gave monthly IM injections and stopped when penile length was equal or >35 mm (8). In their study, boys who underwent PHS <3 months prior to repair had a non-significant higher complication rate (57%) compared to those treated more than 3 months prior (21.7%).

## Adverse Effects

Reported adverse effects such as appearance of pubic hair, aggressive behavior, and increased erections have been reported but are transient and resolve post therapy (4, 6–8, 21). Kaya et al. reported that after topical DHT, 13 of 37 boys experienced pruritus, erythema, and penile pigmentation that normalized after PHS was withdrawn (7). Koff and Jayanthi described dose-related emesis in 1 of 12 boys receiving intramuscular T, leading to cessation of PHS (6). Most studies report no intraoperative complications with a focus on the absence of excess bleeding (2, 21).

In the systematic review, no persistent side effects due to PHS were reported (9). There is some evidence that androgens can induce delay in bone age, increase bone turnover, and increase short-term linear height (25). However, as these concerns have not been supported by evidence in boys with hypospadias treated with PHS at 1-year follow-up, long-term follow-up studies are needed to clearly address these issues (2, 4, 24).

## Our Practice

In our clinical practice, the senior author uses PHS with a single objective criterion – glans size ≤13 mm. Increased glans size is felt to be the single most important variable to make repair less challenging. In a study by Bush et al., they prospectively measured maximum glans width in boys undergoing distal and proximal hypospadias repair, as well as newborns undergoing elective circumcision to determine reference values for penile dimensions (26). A boy undergoing hypospadias repair with a pre-operative glans width of 13 mm typically measured 11 mm after glans wings approximation, correlating to approximately 2 SDs below the normal glans diameter of newborn controls (14.3 mm). Administration of PHS in boys with hypospadias with the objective criteria of ≤13 mm is though to be beneficial to increase glans size within the normal range. Meatus location, penile hypoplasia, and micropenis are felt to be subjective descriptors and penile length is variable secondary to the suprapubic fat pad. Thus, we have been administering PHS to distal, midshaft, and proximal hypospadias and are not selecting patients for PHS subjectively.

Our treatment regimen consists of intramuscular Depo Testosterone Cypionate (100 mg/ml) 2 mg/kg up to three doses (available in Canada). When glans size reaches the typical newborn size of 14 mm, PHS is discontinued. We discontinue PHT at least 4 weeks prior to surgery to decrease the potential risk of excessive intraoperative bleeding secondary to tissue hypervascularity while still maintaining the penile growth effects.

## Conclusion

Androgen stimulation prior to hypospadias repair in some studies has demonstrated a substantial increase in penile length, glans circumference, and tissue vascularity. However, a systematic review suggested that PHS may be related to higher complication rates, and treatment regimens are not standardized. Although PHS is typically well tolerated, there is still a paucity of good data to support clinical guidelines, and well-designed prospective randomized placebo controlled studies are warranted to provide proof of efficacy and further data on safety.

## Conflict of Interest Statement

The authors declare that the research was conducted in the absence of any commercial or financial relationships that could be construed as a potential conflict of interest.
